# Reversing the aging stromal phenotype prevents carcinoma initiation

**DOI:** 10.18632/aging.100318

**Published:** 2011-04-21

**Authors:** Davina A. Lewis, Jeffrey B. Travers, Christiane Machado, Ally-Khan Somani, Dan F Spandau

**Affiliations:** ^1^ Departments of Dermatology, Indiana University School of Medicine, Indianapolis, Indiana; ^2^ Pharmacology and Toxicology, Indiana University School of Medicine, Indianapolis, Indiana; ^3^ Herman B. Wells Center for Pediatric Research, Indiana University School of Medicine, Indianapolis, Indiana; ^4^ Richard L. Roudebush V.A. Medical Center, Indiana University School of Medicine, Indianapolis, Indiana; ^5^ Biochemistry & Molecular Biology, Indiana University School of Medicine, Indianapolis, Indiana

**Keywords:** Senescence, aging, photocarcinogenesis, therapy, UVB

## Abstract

The accumulation of senescent stromal cells in aging tissue changes the local microenvironment from normal to a state similar to chronic inflammation. This inflammatory microenvironment can stimulate the proliferation of epithelial cells containing DNA mutations which can ultimately lead to cancer. Using geriatric skin as a model, we demonstrated that senescent fibroblasts also alter how epithelial keratinocytes respond to genotoxic stress, due to the silencing of IGF-1 expression in geriatric fibroblasts. These data indicate that in addition to promoting epithelial tumor growth, senescent fibroblasts also can promote carcinogenic initiation. We hypothesized that commonly used therapeutic stromal wounding therapies can reduce the percentage of senescent fibroblasts and consequently prevent the formation of keratinocytes proliferating with DNA mutations following acute genotoxic (UVB) stress. Sun-protected skin on the lower back of geriatric human volunteers was wounded by dermabrasion and the skin was allowed to heal for three months. In geriatric skin, we found that dermabrasion wounding decreases the proportion of senescent fibroblasts found in geriatric dermis, increases the expression of IGF-1, and restores the appropriate UVB response to epidermal keratinocytes in geriatric skin. Therefore, dermal rejuvenation therapies may play a significant role in preventing the initiation of skin cancer in geriatric patients.

## INTRODUCTION

Cancer is an age-dependent disease in most mammalian species; in humans over 50% of cancers are found in people over 70 years of age [1]. Recently, the accumulation of senescent cells in aging tissues have been shown to acquire a chronic inflammatory phenotype (called SASP, Senescence-Associated Secretory Phenotype) that serves to promote the growth of initiated tumorigenic epithelial cells [2]. Additional reports have demonstrated that in the skin, the accumulation of senescent fibroblasts also increases the susceptibility of epidermal keratinocytes to carcinogenic initiation [3]. Therefore, while investigations into the role of stromal tissue on the initiation and promotion of cancer are in their infancy, they may serve as potential emerging opportunities for interventional and prophylactic therapeutic strategies [4-6]. Our lab is investigating the role of aging in the development of non-melanoma skin cancer (NMSC) as a model for the effect that aging stromal tissue has in controlling carcinogenesis initiation [3, 6-10]. As such, the skin is an excellent model system for these studies; it is accessible, it has a relevant environmental carcinogen (UVB), and it is possible to easily interrogate human disease.

NMSC has the highest incidence rate of all cancers worldwide, including an estimated 2 million newly diagnosed patients in the United States this year alone [11-12]. Although the mortality of NMSC is relatively low compared to other types of cancer, the morbidity and the cost of treating NMSC is enormous [11, 13]. As NMSC occurs primarily in geriatric individuals, it has been estimated that up to nearly 1% of total Medicare expenses in the United States go towards the treatment of NMSC [13]. Therefore, NMSC is a major burden on our healthcare system [14]. Despite our understanding for decades that sunlight is the main etiologic agent responsible for NMSC (over 90%), the incidence of NMSC continues to rise at an alarming rate [11].

The primary environmental factor that influences the development of skin cancer is exposure to the spectrum of ultraviolet wavelengths found in sunlight. Furthermore, as we age our chances of developing NMSC greatly increases so that at age 65 we have a 50% chance of acquiring a NMSC [15-16]. In fact, 80% of all NMSC are diagnosed in individuals greater than 60 years old [15-16]. While the correlation between aged epidermis and NMSC is apparent, the mechanism responsible for this relationship remains obscure. Early hypotheses describing why the incidence of NMSC increases with age, suggested that excessive sun exposure during adolescence causes mutations in clones of keratinocytes. Subsequently over many decades of genetic selection, these initiated keratinocytes will form detectable tumors [17-18]. However, recent studies have shown that more than 77% of our lifetime sun exposure occurs *after* the age of 18 [19], indicating the vast majority of damaging UVB-irradiation takes place later in life. In fact, more sun exposure occurs after age 59 (26%) than before age 18 (23%) [19]. Recent data from a variety of labs have proposed a modification in the latency theory of carcinogenesis [20-21] based on changes in the effects of stromal cells (i.e. fibroblasts) on epithelial cells in aged individuals [22-23]. This new hypothesis states that the selection of initiated epithelial cells is accelerated in aged tissue due to alterations in gene expression by senescent fibroblasts supporting epithelial cell growth [24-26]. In addition, the aged state of cells may play a greater role in the initiation of carcinogenic DNA mutations than was previously considered [27]. Previously we have shown that the activation of the insulin-like growth factor-1 receptor (IGF-1R) is critical for determining the response of skin keratinocytes to UVB irradiation *in vitro* and *in vivo* [3, 6-10]. If the IGF-1R is functionally inactive *in vitro* at the time of UVB-irradiation, surviving keratinocytes can continue to proliferate with the potential of converting the damaged DNA into initiating carcinogenic mutations [3, 5-6, 10]. Recent data from our laboratories have indicated that similar IGF-1R-dependent UVB responses occur in epidermal keratinocytes *in vivo* [3, 5-6, 10]. Because keratinocytes do not produce IGF-1, the majority of the IGF-1 supplied to the epidermis is produced by dermal fibroblasts. Therefore, any deficiencies in dermal IGF-1 production could have profound effects on the response of epidermal keratinocytes to UVB irradiation. We have demonstrated that such an instance occurs in aged skin, as senescent dermal fibroblasts produce significantly lower levels of IGF-1 than youthful, proliferating fibroblasts [3]. Geriatric skin with lower IGF-1 levels responds inappropriately to UVB exposure and results in the production of keratinocytes that can proliferate with DNA damage. Moreover, we demonstrate that therapeutic treatment of geriatric skin can result in increased levels of dermal IGF-1 and protection against acute UVB-mediated formation of keratinocytes proliferating with DNA damage. We hypothesize that the reduced activation of the IGF-1R in aging skin due to silencing of IGF-1 expression in senescent fibroblasts is an important factor in the dramatic increase in NMSC observed in geriatric patients. The incorporation of recent data from our laboratories and these new ideas on the origins of cancer has led us to a new paradigm to explain non-melanoma skin carcinogenesis [3, 5-6, 10]. This new paradigm indicates that the accumulation of senescent fibroblasts in geriatric dermis leads to a silencing of IGF-1 expression in the skin, resulting in a deficient activation of the IGF-1R in epidermal keratinocytes, causing an inappropriate UVB-response in keratinocytes, leading to proliferating keratinocytes containing DNA mutations, and subsequently photocarcinogenesis [3, 5-6]. Therefore, the susceptibility to develop NMSC is dependent on both the exposure of skin to UVB and the biologic age of the skin.

Given our findings that the lack of endogenous IGF-1 [3] in geriatric skin resulted in an inappropriate pro-carcinogenic response to relatively low doses of UVB [3], and that this inappropriate response was reversed by local injections of *exogenous* IGF-1 [3], these studies have examined the ability of dermal wounding to upregulate *endogenous* IGF-1 levels and restore the appropriate UVB response in geriatric skin. We assayed whether ablation of both the epidermis and papillary dermis by dermabrasion could upregulate IGF-1 expression in geriatric skin and restore the appropriate UVB response. The successful development of the prophylactic therapies as described here could have a major impact on how NMSCs can be prevented in susceptible individuals.

## RESULTS

### Senescent human fibroblasts in vitro contain markers of the DNA damage response

**Figure 1.**
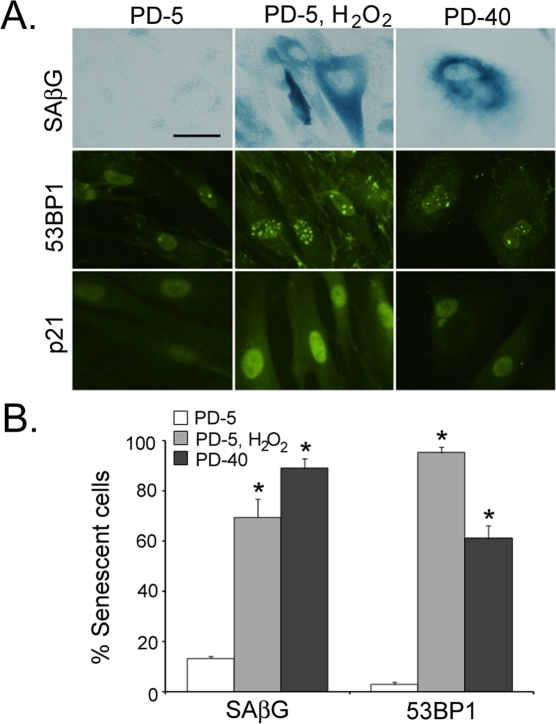
Senescent human fibroblasts contain markers of DNA damage response *in vitro* (**A**) Low passage neonatal normal human fibroblasts (PD-5), stress-induced senescent fibroblasts (PD-5, H_2_O_2_), and replicatively senescent fibroblasts (PD-40) were stained for the presence of senescence-associated β-galactosidase activity (blue), with α-53BP1 antibodies (multiple punctate nuclear staining), or α--p21 antibodies (bar = 20 µm). (**B**) The percentage of senescent cells were determined for senescence-associated β-galactosidase and 53BP1 staining. 53BP1-positive cells contained at least four individual fluorescent pin-point spots per nucleus (asterisks indicate significant difference from PD-5 cells, *p* <0.001, two-tailed t-test).

Normal human fibroblasts that are continually cultured *in vitro* until they reach replicative senescence have historically been identified by their expression of senescence-associated β-galactosidase [28]. Similarly, replicating fibroblasts treated with DNA-damaging chemotherapeutic drugs or pro-oxidative stressors to induce stress-induced senescence have been assayed for senescence-associated β-galactosidase activity to verify their senescence phenotype [3]. However, because identifying senescent cells using senescence-associated β-galactosidase requires an assay of enzymatic activity, its use in specimens from human tissues is not as effective. Recently, it has been described that markers of a DNA-damage response (DDR) are found in most types of senescent cells, whether induced by replication exhaustion, reactive oxygen species, or oncogene expression [29-32]. To determine the reliability of DDR markers to identify senescent fibroblasts in skin, replicating, stress-induced senescent, and replicative senescent fibroblasts were stained by for the traditional senescence-associated β-galactosidase activity, for the presence of 53BP1, and for the expression of cell cycle inhibitor p21 (Fig. 1A). As seen in Fig. 1A, senescent fibroblasts can be identified by nuclei which have greater than four 53BP1-positive foci. When the numbers of senescent fibroblasts were counted in stress-induced senescent and replicatively senescent fibroblasts, the use of either senescent-associated β-galactosidase or 53BP1 foci yielded similar results (Fig. 1B). Therefore, markers of DDR may be useful in identifying senescent fibroblasts *in vivo*.

### Senescent fibroblasts accumulate in geriatric dermis in vivo

**Figure 2.**
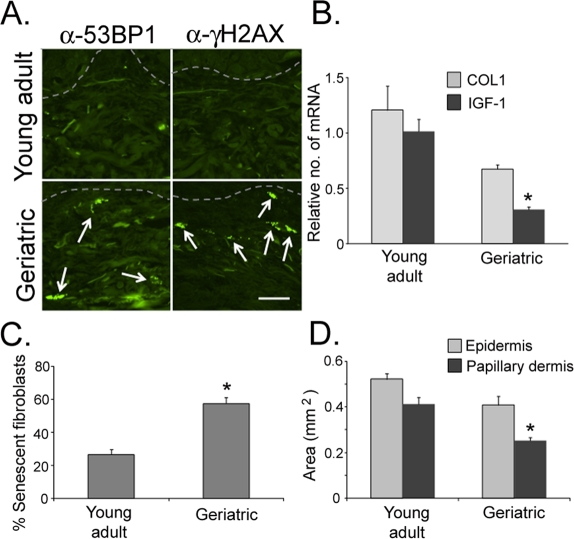
Senescent fibroblasts accumulate in geriatric dermis *in vivo* Biopsies of sun-protected skin were obtained from six young adult (20-28 years old) and six geriatric (>65 years old) volunteers. (**A**) Sections of skin were stained with antibodies to 53BP1 and γH2AX. Positive nuclei are indicated by white arrows; dashed line specifies the location of the basement membrane; bar = 25 µm. (**B**) Quantitative PCR analysis of mRNA isolated from skin biopsies, normalized to actin expression. Asterisk indicate statistical significance from young adult values (IGF-1 *p* = 0.005, COL1 *p* = 0.091; two-tailed t-test). (**C**) The number of senescent fibroblasts (based on circular or elliptical nuclear morphology as determined using Nikon Elements Image Analysis software) was counted in the papillary dermis. Asterisk indicates statistical significance from young adult values (*p* = 0.001; two-tailed t-test). (**D**) The area of the epidermis and papillary dermis were calculated from 3mm punch biopsies using Nikon Elements image analysis software. Asterisk indicates statistical significance from young adult values (Epidermis *p* = 0.0577, Papillary dermis *p* = 0.022; two-tailed t-test).

As we age, the skin becomes altered both phenotypically and biologically. The undulating structure of the dermal-epidermal junction in young skin becomes significantly flattened with age [33-34; Supplemental Fig. 1A]. Both the epidermis and the papillary dermis become atrophied in geriatric skin [33-37; Fig. 2D] and the transcriptome in the geriatric dermis becomes altered, including the relative silencing of the IGF-1 and collagen I genes [3; Fig. 2B]. Additionally, fibroblast morphology transforms from a spindle-shaped cell body and elliptical nucleus to a more rounded cell body and a rounded nucleus [37-38; Supplemental Fig. 1A, white circles]. These morphological changes in fibroblast shape are associated with increasing proportions of senescent fibroblasts in the papillary dermis (Fig. 2C). These senescent fibroblasts can be defined *in vivo* by increased expression of DDR markers (Fig. 2A). Previously, we and others have shown that senescent fibroblasts *in vitro* silence IGF-1 expression [3]. Similarly, intrinsic aging of skin *in vivo* can be characterized by a significantly increased proportion of senescent fibroblasts in the papillary dermis and a corresponding silencing of IGF-1 expression in geriatric dermis.

### Dermabrasion restores young adult function in geriatric dermis

**Figure 3.**
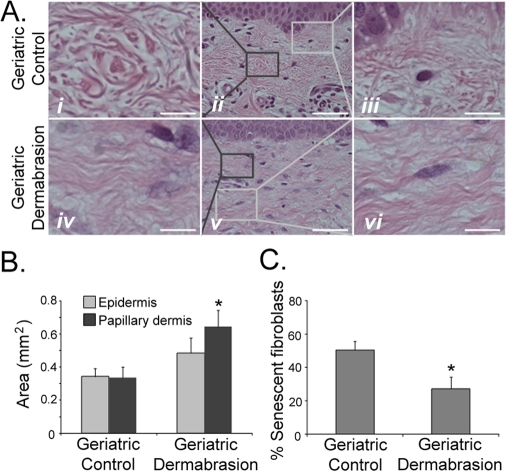
Dermabrasion restores young adult fibroblast function in geriatric dermis A 5 cm^2^ area of sun-protected skin on geriatric (≥65 years old) volunteers was dermabraded. After a healing period of three months, biopsies of untreated and dermabraded skin were obtained. (**A**) Representative H&E sections from untreated and dermabraded geriatric skin. Panels *i* and *iv* are higher magnification images of dark boxes indicated in panels *ii* and *v*. Panels *iii* and *vi* are higher magnification images of light boxes indicated in panels *ii* and *v*. Panels *i* and *iv*, bar = 10 µm; panels *ii* and *v*, bar = 50 µm; panels *iii* and *vi*, bar = 12.5 µm. (**B**) The area of epidermis and papillary dermis were calculated as described in Fig. 2. Asterisk indicates statistical significance from geriatric control values (Epidermis *p* = 0.287, Papillary dermis *p* = 0.013; two-tailed t-test). (**C**) The number of senescent fibroblasts in the papillary dermis was determined as described in Fig. 2. Asterisk indicates statistical significance from control values (*p* = 0.018, two-tailed t-test).

**Figure 4.**
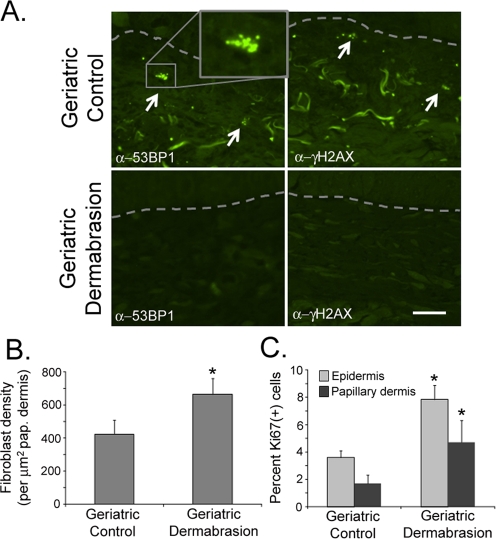
Dermabrasion stimulates fibroblast replication and suppresses senescence in geriatric dermis (**A**) Skin biopsies described in Fig. 3B were stained with antibodies to 53BP1 and γH2AX. Senescent nuclei are indicated by white arrows, the basement membrane is designated by a dashed grey line, bar = 25 µm. (**B**) The density of fibroblasts in the papillary dermis was determined using the Nikon Elements Image Analysis software. Asterisk indicates statistical significance from control values (*p* = 0.0048), two-tailed t-test). (**C**) Sections of biopsies were stained with antibodies to Ki67. The percentage of Ki67(+) fibroblasts in the papillary dermis and the percentage of Ki67(+) keratinocytes in the basal layer of the epidermis were calculated using Nikon Elements image analysis software. Asterisks indicate statistical significance from control values (papillary dermis, *p* = 0.039; epidermis p = 0.058, two-tailed t-test).

Cosmetic dermal rejuvenation techniques have been widely used to stimulate the production of new collagen synthesis by inducing a ‘wounding response' in the skin [39-40]. As such, it was of interest to determine if these dermal rejuvenation techniques restored a more youthful phenotype and biology to geriatric skin, and specifically to determine if these techniques could restore the appropriate DNA-damage response found in young skin to UVB-irradiated geriatric skin. Small areas of sun-protected skin on geriatric volunteers were treated by dermabrasion. Biopsies of dermabraded and untreated skin were analyzed after the treated sites were allowed to heal for three months. Consistent with previous reports, dermabraded skin demonstrated increased synthesis of collagen [39-40; Supplemental Fig. 2] and a restoration of the dermal collagen structure similar to that found in young adults (Fig. 3A, panels *i* and *iv*). Dermabasion also reversed the aging-associated atrophy of the papillary dermis (Fig. 2D) by significantly increasing the area of the papillary dermis (Fig. 3B). The thickness of the epidermis was modestly increased by dermabrasion, although this result was not statistically significant (Fig. 3B). Increased thickness of the papillary dermis was accompanied by an increase in fibroblast density in the dermabraded geriatric skin (Fig. 4B; see Supplemental Fig. 3 for example of fibroblast verification) and statistically greater numbers of replicating keratinocytes and fibroblasts (Fig. 4C). The increased proliferative potential of fibroblasts in the dermis corresponded with a decrease in the proportion of dermal senescent fibroblasts (Fig. 3C). The round phenotype of the senescent fibroblast nuclei in control geriatric dermis was replaced with increasing percentages of replicating elliptical fibroblast nuclei (Fig. 3A, panels *iii* and *vi*). The loss of senescent cells in dermabraded skin can also be observed by assaying for DDR markers. In contrast to the abundant expression of DDR markers in senescent fibroblasts of control geriatric dermis, DDR-positive fibroblasts are not detected in dermabraded geriatric dermis (Fig. 4A).

**Figure 5.**
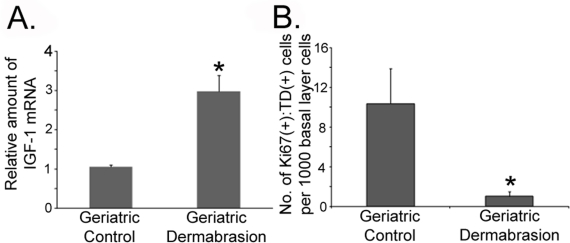
Dermabrasion upregulates IGF-1 expression and restores the appropriate UVB response to geriatric skin Small areas of dermabraded and untreated skin on geriatric volunteers described in Fig. 2 were irradiated with UVB (dose of 350 J/m^2^). Twenty-four hours post-UVB, the irradiated skin was biopsied. (**A**) Total mRNA was isolated and the relative level of IGF-1 expression was determined by quantitative PCR (normalized to actin expression). Asterisk indicates statistically significant differences from control values (*p* <0.006, two-tailed t-test). (**B**) Sections of biopsies were stained with antibodies to Ki67 and thymine dimers (TD). The number of dual Ki67(+):TD(+) basal keratinocytes were determined for both sets of biopsies. Asterisk indicates statistically significant differences from control values (*p* <0.05, two-tailed t-test).

At three months following dermabrasion, IGF-1 expression was three-fold higher in treated dermis compared to geriatric controls (Fig. 5A). To assay the UVB-response, small areas of the dermabraded skin and untreated skin on the opposing hip/buttock were irradiated with 350 J/m^2^ (one MED) of UVB. Twenty-four hours post-irradiation (the time required to clear TD lesions), the UVB-irradiated areas were biopsied and the basal layer keratinocytes were assayed for co-expression of proliferation (Ki67) markers and UVB-induced DNA damage (TD; see examples of scored keratinocytes in Supplemental Figure 4). Consistent with the ability of dermabrasion to upregulate IGF-1 levels, UVB irradiation of control versus dermabraded skin were similar to our findings using young skin or geriatric skin treated with exogenous IGF-1 (i.e., no keratinocytes proliferating with DNA damage in the dermabraded skin in contrast to significant numbers of Ki67+:TD+ basal keratinocytes in untreated UVB-irradiated skin; Fig. 5B). These findings indicate that dermal rejuvenation upregulates IGF-1 levels and normalizes the UVB response in geriatric skin.

## DISCUSSION

We have demonstrated *in vivo* that geriatric skin accumulates increasing proportions of senescent fibroblasts as measured by changes in cellular and nuclear morphology [3; Fig. 3] as well as the induction of DNA-damage recognition proteins associated with cellular senescence [3; Fig. 3]. Furthermore, we have shown that geriatric skin silences IGF-1 expression [3] leading to deficient activation of the IGF-1R [3] on geriatric epidermal keratinocytes. Therefore, when geriatric skin is irradiated with UVB, a portion of the epidermal keratinocytes respond inappropriately by allowing replication of UVB-damaged DNA and potentially creating ‘initiated' tumor cells [3, 5-6]. The role of IGF-1 *in vivo* was confirmed by its ability to correct this inappropriate UVB response in geriatric skin by injection of IGF-1 into the dermis prior to UVB irradiation [3]. Thus, therapies that can restore IGF-1 expression to levels seen in young adult dermis could potentially prevent the initiation of carcinogenesis in geriatric skin. Skin rejuvenation techniques, including dermabrasion, have been widely used to stimulate dermal collagen production and promote a youthful appearance of the skin. We found that dermabrasion of sun-protected geriatric skin decreased the proportion of senescent fibroblasts resulting in increased IGF-1 expression. Most importantly, dermabrasion corrected the inappropriate UVB response normally observed in geriatric skin. These results suggest that dermabrasion of geriatric skin can prophylactically prevent non-melanoma skin carcinogenesis.

It is interesting to note that the use of dermabrasion to prophylactically treat actinic keratosis and NMSC was described over 40 years ago [41]. A number of reports have demonstrated that dermabrasion can reduce the incidence of actinic keratosis and NMSC up to 95% in susceptible individuals for many years after treatment [42-44]. However, the use of dermabrasion has fallen out of favor as a primary method for the prophylaxis of NMSC despite the fact that newer methods of prophylactic therapy, i.e. lasers, topical chemotherapy (5-fluorouracil, imiquimod), have never achieved the same level of effectiveness as dermabrasion [45-49]. In fact, studies of the efficacy of these modalities often use dermabrasion as the gold standard for NMSC prophylaxis [50].

Although the early studies on dermabrasion demonstrated its success in NMSC prophylaxis, the mechanism of how it prevented NMSC was unclear. It was hypothesized (but unproven) that the effectiveness of dermabrasion was due to the removal of previously initiated carcinogenic keratinocytes. However, if this hypothesis was true, other ablative procedures should be just as successful in treating NMSC, but they are not. Our studies suggest a new mechanism by which dermabrasion can prevent NMSC carcinogenesis which focuses on its effect on dermal fibroblasts. The accumulation of senescent fibroblasts in geriatric dermis alters the susceptibility of epidermal keratinocytes to accumulate and fix UVB-induced mutations in their genomes. Furthermore, senescent fibroblasts have been shown to provide an enhanced environment for the growth of carcinogenically initiated epithelial cells via their upregulation of inflammatory cytokines [22-24]. Therefore, the preponderance of senescent fibroblasts in geriatric dermis not only promotes initiating mutations in UVB-exposed keratinocytes but they also promote the expansion of initiated clones of keratinocytes. As demonstrated in these studies, dermabrasion can dramatically reduce the proportion of senescent fibroblasts in treated geriatric dermis. Importantly, the elimination of senescent fibroblasts restores the expression of IGF-1 to normal levels, increases the production of collagen, and prevents the inappropriate UVB response in epidermal keratinocytes. These studies demonstrate an alternative mechanism, other than just removal of initiated keratinocytes, by which dermabrasion can protect geriatric skin from actinic neoplasia.

## METHODS

### Human subjects

Geriatric volunteers were recruited from patients treated at Indiana University dermatology clinics. These studies were approved by the Indiana University School of Medicine Institutional Review Board and subjects have signed approved consent forms. Specific requirements for inclusion/exclusion from these studies can be found in the Supplemental Materials.

### Dermabrasion

Prior to treatment, a region of sun-protected hip/buttock skin was photographed. Next, an approximately 5 × 5 cm area of the subject's lower hip/buttock skin was isolated and anesthetized with xylocaine anesthesia. Under sterile conditions the localized area of skin was then abraded with sterile, coarse (#60) sandpaper down to the mid dermis, with complete removal of all epidermis and superficial dermis. The wounded area was bandaged with moist, occlusive dressings and the volunteer was instructed to change the dressing twice daily until the wound is re-epithelized in 1-2 weeks. Approximately three months later (˜Day 90 +/− 7 days) the volunteer returned to the clinic and a localized area 1 × 1 cm of either dermabrasion or untreated normal skin on the opposite hip/buttock was irradiated with dose of 350 J/m^2^ of UVB. In Fitzpatrick Skin Types I and II, this dose of UVB is sufficient to cause a minimal erythematous reaction. Permanent marker was used to outline the areas of skin that was irradiated. Twenty-four hours following UVB exposure, photographs were taken of the skin to document the extent of the UVB reaction. The irradiated skin, as well as unirradiated adjacent skin, was removed by punch biopsy, (4 mm punch biopsies of the UVB-treated skin and 3 mm punch biopsies of unirradiated skin; 4 biopsies per individual).

### Human UVB response assay

The epidermal response to UVB irradiation was assayed as previously described [3]. Briefly, thin paraffin-embedded sections from unirradiated and UVB-irradiated biopsies were simultaneously stained with antibodies to Ki67 and thymine dimers. Secondary antibodies that specifically detect only one of the primary antibodies are conjugated to the fluorescent dyes AlexaFluor 488 (detecting Ki67, emitting green wavelengths), and AlexaFluor 568 (detecting thymine dimers, emitting red wavelengths). Images were captured sequentially along the entire length of the biopsy specimen (3mm non-irradiated, 4mm irradiated) using a Nikon Eclipse 80i microscope with Intensilight epifluorescence. These images were analyzed by counting the number of keratinocytes in contact with the basement membrane that are Ki67(+), thymine dimer(+), and Ki67(+):thymine dimer(+). These numbers were expressed as a percentage of total basal layer keratinocytes in the biopsy specimen (determined by counting basal layer keratinocytes for each specimen on H&E-stained slides).

### Statistical analysis

Statistical analyses were done by two-tailed Student's t test. Statistical significance was defined as p < 0.05 unless otherwise noted in the figure legend.

### Supplemental Material

Four additional figures and specific protocols for quantitative reverse-transcription PCR, immunofluorescence, growth of human fibroblasts, and senescence-associated β-galactosidase assays can be found in the Supplemental Material.

## SUPPLEMENTARY MATERIALS


