# Supplementary material for: Reversing the aging stromal phenotype prevents carcinoma initiation
Source: Aging (Albany NY). 2011 Apr 21;3(4):407–16. doi: 10.18632/aging.100318 (PMC3117456; doi:10.18632/aging.100318)
Supplement: Supplementary file 1 [file aging-03-407-s001.pdf]

SUPPLEMENTAL MATERIAL

Supplemental Experimental Procedures

Quantitative Reverse-Transcriptase PCR. Homogenized tissue from dissected dermal sections was lysed using RNeasy kit (Qiagen) buffers. Cell lysates were then further homogenized using Shredder columns (Qiagen) and RNA isolation continued with RNeasy kit. All of the reagents used for RT and PCR are obtained from SuperArray Biosciences, Frederick, MD. The following were added to a 0.2 ml tube where first a genomic DNA elimination step was performed on 2 µg RNA total volume 10 µl was heated to 42°C for 5 minutes and chilled on ice. Next the reverse transcription cocktail was prepared and 10 µl added to the RNA; 2 µl RT enzyme mix, 4 µl RT buffer, 1 µl primer and external control mix, 3 µl RNase free water. Mixture was heated 42°C for 15 minutes, 95°C for 5 minutes and chilled on ice for experiments, the final volume of cDNA was 20 µl. qRT-PCR is performed using a LightCycler PCR (Roche Scientific, Fishers IN). Quantization of experimental qRT-PCR products was determined by comparison with external control qRT-PCR products from templates of a known copy number. Relative copy numbers of experimental mRNA are then determined following adjustment with actin controls from the same tissue.

Immunofluorescence. Paraffin-embedded sections were deparaffinized, hydrated, and rinsed with tris-buffered saline with tween 20 (TBS) (DAKO, Carpenteria, CA). Antigen retrieval was performed using a water bath at 95°C for 20 minutes with DAKO Target Retrieval buffer. After cooling, the slides are rinsed with TBS, and the slides were then transferred to a clean 100 mm bacterial glass petri dish containing PBS-saturated filter paper under a strip of Parafilm. Primary antibodies (1:50) diluted in 3% BSA in TSB was added to the tissues. The lid was placed on the petri dish and the coverslips incubated for 1 hour at room temperature. The tissues were rinsed in PBS (three 10 minutes washes), and then the appropriate secondary antibody conjugated to the desired fluorochrome was added for 30 minutes at room temperature in the dark. The sections were washed as before, the edges blotted dry, and then mounted with coverslips using Fluoromount G. Antibodies used included: α-53BP1 (Abcam, Cambridge, MA), α-p21 (Cell Signaling, Danvers, MA), α-γH2AX (Millipore, Temecula, CA), α-prolyl-4-hydroxylase (Millipore, Temecula, CA), α-Ki67 (NeoMarkers, Freemont, CA), and α-thymine dimers (Kamiya Biomedical, Seattle, WA).

Senescence-associated β-galactosidase assays. Fibroblasts were washed twice with PBS and fixed with 2% formaldehyde/0.2% glutaraldehyde at room temperature for 10 minutes. After two additional washes with PBS, 2 ml of staining solution (150 mM sodium chloride, 25.2 mM sodium phosphate dibasic, 7.36 mM citric acid, 5 mM potassium ferricyanide, 5 mM potassium ferrocyanide, 2 mM magnesium chloride, 1 ng/ml 5-bromo-4-chloro-3-indolyl-□-D-galactoside, pH 6.0)[25], were added to the cells and they were incubated at 37°C overnight. The cells were again washed with PBS and photographed by bright field microscopy to count blue cells and phase contrast microscopy to count total cells. At least four fields (100X magnification, approximately 200 cells/field) were counted for each plate of cells; at least two plates of cells for each condition (or cell type) were assayed in each experiment.

Isolation and culture of normal human fibroblasts. Excised foreskin tissue was washed with antibiotics, the tissue minced, and individual cells released from the tissue by trypsin digestion (8). Keratinocytes and fibroblasts were separated by differential resistance to treatment with EDTA. Fibroblasts were grown in Dulbecco's Modified Eagles medium containing 10% fetal calf serum. All relevant procedures using human tissue have been approved by the Indiana University School of Medicine Institutional Review Board.

SUPPLEMENTAL FIGURES

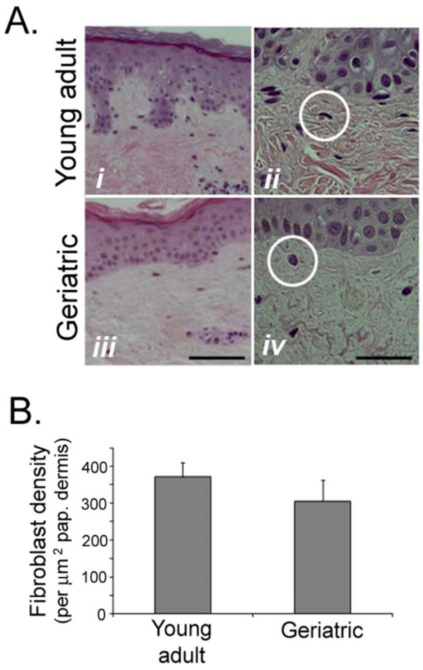

Supplementary Figure 1. Phenotypic changes in geriatric skin. (**A**) H&E sections of the biopsies described in Fig. 2. White circles indicate elliptical nucleus of a replicating fibroblast in panel *ii* and the spherical nucleus of a senescent fibroblast in panel *iv*. Bar=100 μm in panels *i* and *iii*; bar=25 μm in panels *ii* and *iv*. (**B**) The density of fibroblasts was determined in young adult and geriatric skin as described in Fig. 2 (p=0.093; two-tailed t-test).

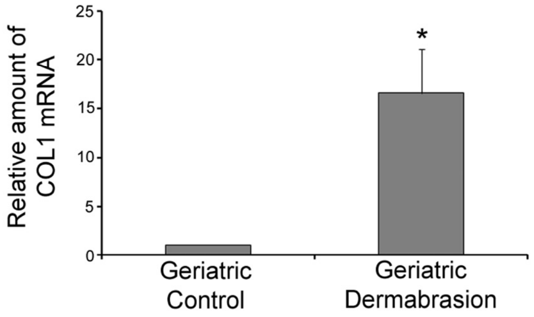

Supplementary Figure 2. Dermabrasion increases collagen expression. Quantitative PCR was conducted on biopsies described in Fig. 2. The relative amount of collagen I mRNA (normalized to actin expression) is shown. Asterisk indicates statistical significance from young adult values (p=0.018,two-tailed t-test).

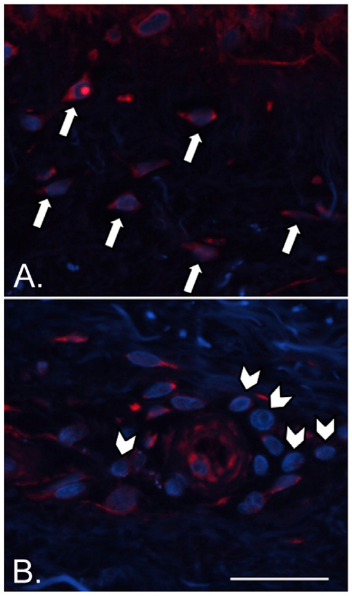

Supplementary Figure 3. Identification of dermal fibroblasts in geriatric dermis. Example of how the identity of fibroblasts was confirmed in the papillary dermis. Sections were stained with antibodies to α-prolyl-4-hydroxylase (red) and DAPI (nuclear-specific stain, blue). Fibroblasts stain positive for α-prolyl-4-hydroxylase and are indicated by arrows in panel **A**. Non-fibroblasts are indicated in panel **B** by chevrons.

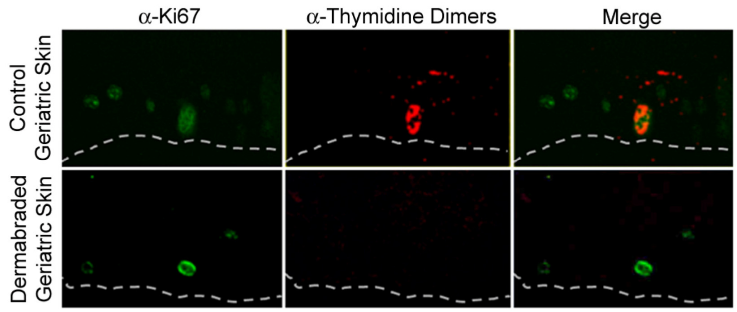

Supplementary Figure 4. Example of the UVB-response assay. Two cm^2 areas on the lower backs of volunteers were irradiated with UVB (350 J/m^2). Twenty-four hours following irradiation, a four mm punch biopsy was obtained from the irradiated skin. Sections of formalin-fixed, paraffin-embedded tissue were stained with both α-Ki67 (staining green) and α-thymine dimer antibodies (staining red). The merged images are shown to the right of the figure. The top series of panels is an example of a cell positive for both Ki67 and thymine dimers (staining yellow, inappropriate UVB response) while the cell indicated in bottom panels is only positive for Ki67 (staining green, appropriate UVB response). The location of the basement membrane is indicated by a grey dashed line. Similar sections were stained with H&E to determine the total number of basal layer keratinocytes in each biopsy.
